# Tunable Resistive Switching Behaviors and Mechanism of the W/ZnO/ITO Memory Cell

**DOI:** 10.3390/molecules28145313

**Published:** 2023-07-10

**Authors:** Zhiqiang Yu, Jinhao Jia, Xinru Qu, Qingcheng Wang, Wenbo Kang, Baosheng Liu, Qingquan Xiao, Tinghong Gao, Quan Xie

**Affiliations:** 1 Faculty of Electronic Engineering, Guangxi University of Science and Technology, Liuzhou 545006, China; 221077051@stdmail.gxust.edu.cn (J.J.); 221068387@stdmail.gxust.edu.cn (X.Q.); 221076997@stdmail.gxust.edu.cn (Q.W.); 221077056@stdmail.gxust.edu.cn (W.K.); liubaosheng@gxust.edu.cn (B.L.); 2 Institute of Advanced Optoelectronic Materials and Technology, College of Big Data and Information Engineering, Guizhou University, Guiyang 550025, China; qqxiaohn@126.com (Q.X.); gaotinghong@sina.com (T.G.); qxie@gzu.edu.cn (Q.X.); 3 Wuhan National Laboratory for Optoelectronics, School of Optical and Electronic Information, Huazhong University of Science and Technology, Wuhan 430074, China

**Keywords:** sol–gel, ZnO nanofilms, nonvolatile, multilevel, oxygen vacancies

## Abstract

A facile sol–gel spin coating method has been proposed for the synthesis of spin-coated ZnO nanofilms on ITO substrates. The as-prepared ZnO-nanofilm-based W/ZnO/ITO memory cell showed forming-free and tunable nonvolatile multilevel resistive switching behaviors with a high resistance ratio of about two orders of magnitude, which can be maintained for over 10^3^ s and without evident deterioration. The tunable nonvolatile multilevel resistive switching phenomena were achieved by modulating the different set voltages of the W/ZnO/ITO memory cell. In addition, the tunable nonvolatile resistive switching behaviors of the ZnO-nanofilm-based W/ZnO/ITO memory cell can be interpreted by the partial formation and rupture of conductive nanofilaments modified by the oxygen vacancies. This work demonstrates that the ZnO-nanofilm-based W/ZnO/ITO memory cell may be a potential candidate for future high-density, nonvolatile, memory applications.

## 1. Introduction

As the fourth fundamental element, the memristor has been regarded as one of the most potential candidates for future nonvolatile memory devices due to its merits in terms of non-volatility, fast memory speed, high integration density, good endurance, long retention, ultra-low power dissipation, and multilevel behaviors [1,2,3,4,5,6,7,8,9,10,11,12,13,14,15,16,17,18,19,20,21,22,23,24,25,26,27,28,29,30,31,32,33,34,35,36,37,38,39,40,41,42,43,44,45,46,47,48,49,50,51,52,53,54,55,56,57,58,59,60]. In recent decades, various oxides have acted as the dielectric materials for memristor applications. Among them, the transition metal oxides, such as ZnO [3,4,5,6,7], TiO_2_ [8,9,10,22,56,58,59,60], HfO_2_ [11], GaO_x_ [12], α-Fe_2_O_3_ [13], Co_3_O_4_ [14], CuO_x_ [15,23], WO_3_ [16], NiO [17], In_2_O_3_ [18], TaO_x_ [19], and CeO_2_ [20] have become a new focus because of their excellent resistive switching performances. In recent decades, increasing interest has been paid to zinc oxide (ZnO)-based devices because of their features including non-toxicity, suitable band gap (3.37 eV), high electron mobility (~120 cm^2^ Vs^−1^), large exciton-binding energy (60 meV), and small electron-hole collision ionization coefficient [21]. The third-generation semiconductor material ZnO has shown excellent potential for nonvolatile memory applications. In particular, ZnO nanomaterials, especially those involving semiconducting and metal oxide nanomaterials corresponding to ZnO nanowires [5,27,32,38,48,50,55], ZnO nanotubes, and ZnO nanofilms [7,21,24,28,29,30,34,36,39,40,43,44,46,49,52,53,54], have recently received remarkable attention for different nano-electronic and optoelectronic applications due to their unique chemical and physical properties inherently different from the ZnO bulk materials, which rely mainly on their unique shape and size. Recently, the transition metal oxide ZnO nanofilms with their simple chemical composition, rich reserves, nontoxicity, and since they contain regulated oxygen vacancies, they have been regarded as one of the most outstanding oxide materials due to their potential applications in future nonvolatile resistive memory devices.

To achieve the high-density nonvolatile memory applications of memristors, the most fundamental solution is to reduce the device size itself [22,57,61,62]. Another effective strategy is to utilize the controllable multilevel resistive switching properties of the device. In addition, the electroforming process is normally required to induce reliable resistive switching behavior owing to the low density of oxygen vacancy defects in pristine-state oxides [23,38], which is an obstacle for commercial device applications. Thus, increasing attention has been devoted to the forming-free and multilevel resistive properties of nanostructured memristors for high-density nonvolatile memory applications. In recent decades, various ZnO-based memristors have shown excellent resistive switching properties for nonvolatile memory applications arising from the intrinsic defects related to oxygen vacancies in the ZnO layer [3,4,5,6,7,24,25,26,27,28,29,30,31,32,33,34,35,36,37,38,39,40,41,42,43,44,45,46,47]. In addition, several resistive switching mechanisms such as the formation and rupture of conducting filaments [7,21,24,27,28,29,30,32,39,44,46,48,52,53,54,55], the trap-controlled space-charge-limited conduction mechanism [34,40,43], the Arrhenius activation theory [38], the Schottky emission [36,49], electron tunneling [5], and the valence change mechanisms [50] have been developed to explain the nonvolatile resistive switching behaviors of ZnO-nanofilm-based memory devices.

Recently, many preparation methods including the spin coating technique [5,46], the magnetron sputtering method [21,30,43,44,49,52,53], chemical vapor deposition [24,27,50,55], the hydrothermal method [32,48], the pulsed laser deposition [34,36], the dip coating method [38,54], and the co-precipitation method [51] have been performed to prepare ZnO-nanofilm-based memory devices. Among them, the spin coating technique is suitable for the controlled synthesis of the large-scale ZnO nanofilms due to its low cost, simple process, and relatively mild reaction conditions; it is one of the effective methods for preparing the ZnO nanofilms. Additionally, the tunable nonvolatile multilevel resistive switching performances have been achieved by modulating the compliance currents [3,7,25] and reset voltages [31,39] of the ZnO-based memristors. However, there have been no such reports about the synthesis and nonvolatile resistive switching properties of ZnO-nanofilm-based W/ZnO/ITO memory cells so far. Moreover, a proper mechanism to illustrate the forming-free and nonvolatile multilevel resistive switching behaviors of ZnO-nanofilm-based W/ZnO/ITO memory cells is still urgently desired.

Herein, a facile sol–gel spin coating method has been proposed for the synthesis of spin-coated ZnO nanofilms on ITO substrates. The forming-free and tunable nonvolatile multilevel resistive switching phenomena of the ZnO-nanofilm-based W/ZnO/ITO memory cell with a relatively higher resistance ratio of about two orders of magnitude were achieved by modulating different set voltages. The filamentary resistive switching mechanism modified by oxygen vacancies has been proposed to interpret the nonvolatile resistive switching performances of the W/ZnO/ITO memory cell. This work suggests that the ZnO-nanofilm-based W/ZnO/ITO memory cell may be a potential candidate for high-density nonvolatile memory applications.

## 2. Results and Discussion

**Figure 1:**
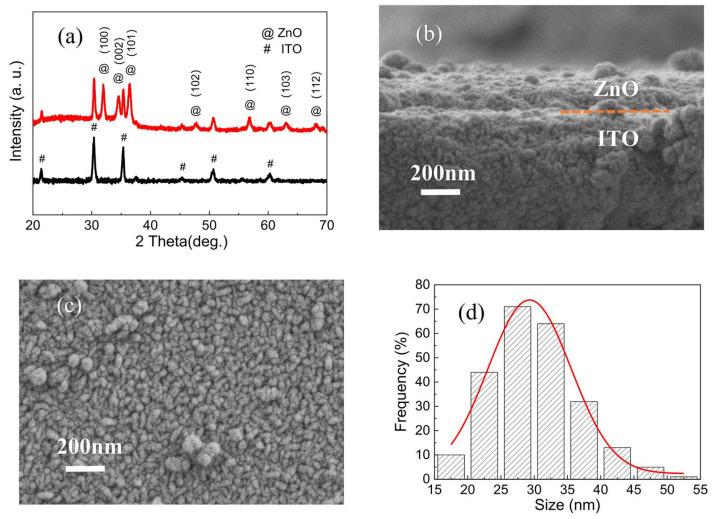
(**a**) XRD pattern of the ZnO nanofilms. FESEM images of the ZnO nanofilms: (**b**) the cross-sectional FESEM image, (**c**) the high magnification, top-view FESEM image. (**d**) The size distribution histogram of ZnO nanograins.

Figure 1a displays the XRD pattern of the as-prepared ZnO nanofilms. All the XRD diffraction peaks in the ZnO nanofilm spectrum are indexed to the hexagonal (wurtzite) phase (JCPDS Card No. 36-1451) [5]. The presence of the strong peaks suggests the high crystal quality of the ZnO nanofilms. In particular, the significantly stronger XRD diffraction peaks such as (100), (002), and (101) imply that the as-prepared wurtzite ZnO nanofilms are highly aligned along the c-axis, which are perpendicular to the ITO substrate. Figure 1b shows the cross-sectional FESEM image of the as-prepared ZnO nanofilms. The as-prepared ZnO nanofilms with a thickness of about 90 nm were surveyed, which can be acted as the dielectric material of the ZnO-nanofilm-based W/ZnO/ITO memory cell. Figure 1c presents the top-view FESEM images of the as-prepared ZnO nanofilms at high magnification. It was found that the as-prepared ZnO nanofilms are comprised of ZnO nanograins, and the nanograin distribution on the entire surface of the ZnO nanofilms was relatively uniform. Furthermore, the size distribution histogram of the ZnO nanograins, as shown in Figure 1d, further indicates that the mean nanograin size of the ZnO nanofilms was approximately 29.3 nm.

The mean crystal size of the as-prepared ZnO nanofilms can be evaluated by the Scherer’s equation [47] as follows:

(1) $$D=\frac{0.9\lambda }{\beta cos\theta }$$

where D is the crystal size of the ZnO nanofilms, β is the full width at half-maxima, λ is the X-ray wavelength (1.5406 Å), and θ is the diffraction angle. The mean crystal size of the ZnO nanofilms was calculated to be 25 nm, which demonstrates the nanocrystal feature of the as-prepared ZnO nanofilms composed of nanograins.

**Figure 2:**
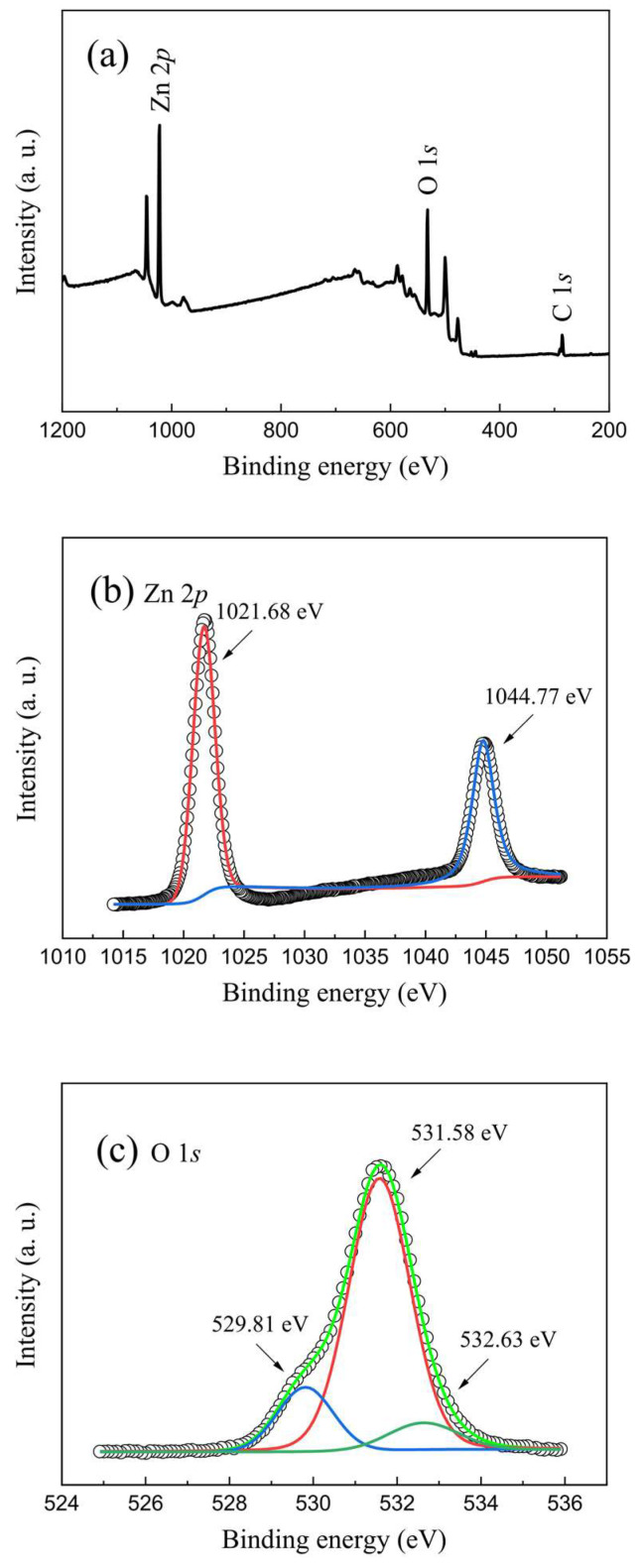
(**a**) Survey XPS spectra of the as-prepared ZnO nanofilms. (**b**) Zn 2*p* and (**c**) O 1*s* high-resolution XPS spectra of the as-prepared ZnO nanofilms.

Figure 2 shows the survey XPS spectra, the Zn 2*p* and the O 1*s* high-resolution XPS spectra of the as-prepared ZnO nanofilms near the surface. It was observed that all the Zn and O elements, together with the C elements, can be observed in the as-prepared ZnO nanofilms, where the C elements stem from the carbon source in the air adsorbed onto the surface of the as-prepared ZnO nanofilms, which was employed to calibrate the other elements including the Zn and O elements, as shown in Figure 2a. Figure 2b depicts the Zn 2*p* high-resolution XPS spectra and the corresponding Gaussian fitting peaks of the as-prepared ZnO nanofilms. The Zn 2*p* XPS peaks observed at 1044.4 eV and 1021.3 eV binding energies can be ascribed to the Zn 2*p*_1/2_ and Zn 2*p*_3/2_, respectively. The spin-orbit splitting binding energy between the Zn 2*p*_1/2_ and Zn 2*p*_3/2_ was evaluated to be 23.1 eV, which suggests the presence of Zn^2+^ in the ZnO nanofilms [5,21]. The asymmetric O 1*s* XPS spectra, as shown in Figure 2c, can be deconvoluted with three Gaussian fitting peaks located at 529.2 eV, 530.8 eV, and 531.9 eV binding energies, which correspond to the lattice oxygen, oxygen vacancies, and the chemisorbed oxygen groups [37], respectively. In particular, the relative concentration of oxygen vacancies has been calculated to be 76.4% from the peak area around 530.8 eV, which is higher than that of the chemisorbed oxygen groups (8.1%) around 531.9 eV in the as-prepared ZnO nanofilms. The fitting results of the XPS spectra imply that a significant amount of oxygen vacancies exist in the as-prepared ZnO nanofilms, which can act as the trapping center and be responsible for the tunable nonvolatile multilevel resistive switching performances of the ZnO-nanofilm-based W/ZnO/ITO memory cell.

**Figure 3:**
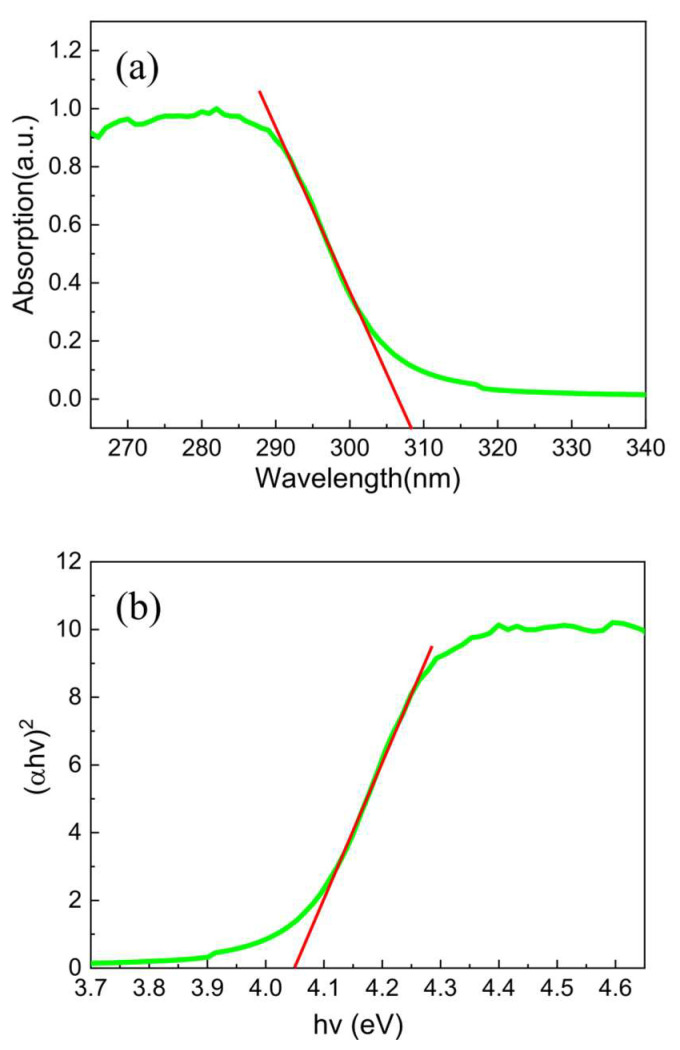
(**a**) UV–visible absorption spectra and (**b**) the Tauc plots of the ZnO nanofilms.

Figure 3a,b show the UV–visible absorption spectra and the corresponding Tauc plots of the as-prepared ZnO nanofilms, respectively. It is appreciable that the as-prepared ZnO nanofilms composed of nanograins displayed an excellent optical absorption capability with an absorption edge of about 308 nm in the UV absorption region, as shown in Figure 3a. Furthermore, the optical band gap $E_{g}$ of the as-prepared ZnO nanofilms can be obtained by the following Tauc equation [42]:

(2) $$\;{\left(\alpha h\upsilon \right)}^{n}=A\left(h\upsilon -E_{g}\right)\;$$

where α is the absorbance coefficient of the as-prepared ZnO nanofilms, h is the Planck’s constant, v is the vibration frequency, and A is the optical constant. Furthermore, *n* is the vibration frequency of ZnO (*n* = 2) due to its direct band gap. As shown in Figure 3b, the optical band gap of the ZnO nanofilms composed of nanograins was be found to be 4.05 eV, which is larger than that of the pristine ZnO bulk [21] (3.37 eV) due to the nanometer size effect, further confirming the nanocrystal nature of the wurtzite ZnO nanofilms composed of nanograins.

**Figure 4:**
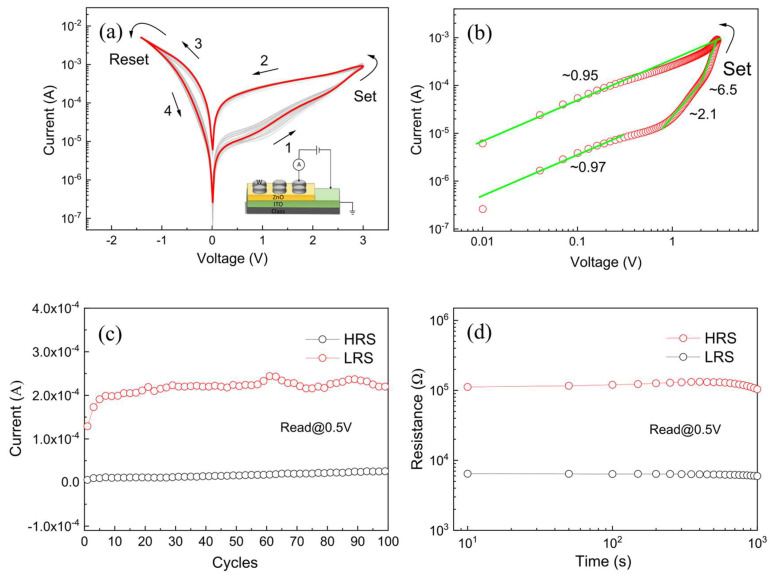
(**a**) The semi-logarithmic *I*-*V* curves of the W/ZnO/ITO memory cell for 100 successive cycles; inset is the schematic configuration of the device. (**b**) The double-logarithmic *I*-*V* curve of the device. (**c**) Endurance performance of the device. (**d**) Retention test of the device.

In order to evaluate the nonvolatile resistive switching behavior of the as-prepared ZnO-nanofilm-based W/ZnO/ITO memory cell, the typical *I*-*V* measurements of the ZnO-nanofilm-based W/ZnO/ITO memory cell were carried out by sweeping the applied voltage in the sequence of 0 V → +3 V → 0 V → −1.5 V → 0 V with a compliance current fixed at 5 mA to protect the device from irreversible breakdown during the tests, as shown in Figure 4. Figure 4a shows the semi-logarithmic *I*-*V* curves of the ZnO-nanofilm-based W/ZnO/ITO memory cell over 100 successive cycles. The arrows and numbers as recorded in Figure 4a present the applied voltage sweeping direction and sequence in the device, respectively. It was observed that the ZnO-nanofilm-based W/ZnO/ITO memory cell showed a stable nonvolatile and forming-free bipolar resistive switching behavior. Moreover, the asymmetric *I*-*V* curves corresponding to the low forward and high reverse currents in the low resistance state (LRS) also revealed the self-rectifying feature of the W/ZnO/ITO memory cell. As shown in Figure 4a, the pristine resistance state of the W/ZnO/ITO memory cell was the high resistance state (HRS). When the applied voltage increased from 0 V to +3 V, the ZnO-nanofilm-based W/ZnO/ITO memory cell will gradually transition from the HRS to LRS and the set process occurred at +3 V (V_set_). Subsequently, the ZnO-nanofilm-based W/ZnO/ITO memory cell will preserve the LRS until the applied voltage decreases to −1.5 V (V_reset_), which means that the reset process occurs at V_reset_ and the device switches from the LRS to the pristine HRS, indicating the nonvolatile bipolar resistive switching behavior of the as-prepared ZnO-nanofilm-based W/ZnO/ITO memory cell.

To further illustrate the nonvolatile resistive switching mechanism of the as-prepared ZnO-nanofilm-based W/ZnO/ITO memory cell, we plotted the double-logarithmic *I*-*V* curve of the device (Figure 4b). In the LRS region, the linear *I*-*V* curve with a slope of 0.95 suggests an Ohmic conduction feature (I∝V) of the ZnO-nanofilm-based W/ZnO/ITO memory cell. In the HRS region, the slope of the *I*-*V* curve changed from 0.97 to 2.1, and then to 6.5 with rising applied voltage. The *I*-*V* response in the HRS can be divided into three different regions corresponding to the Ohmic conduction region (I∝V), the Child’s law conduction region ($I\propto V^{2}$), and the trap-filled limited conduction region ($I\propto V^{m},\;m>2$), demonstrating the trap-controlled space-charge-limited current (SCLC) performance [4] of the ZnO-nanofilm-based W/ZnO/ITO memory cell in the HRS. During the set process, a gradual jump of current from the HRS to LRS occurred at V_set_, which corresponds to the transition from the SCLC conduction region to the Ohmic conduction region of the ZnO-nanofilm-based W/ZnO/ITO memory cell. Significantly, the nonvolatile resistive switching behavior of the as-prepared ZnO-nanofilm-based W/ZnO/ITO memory cell might be assigned to the filamentary resistive switching mechanism.

In the Ohmic conduction region, the current density $\;J_{o}$ can be given as

(3) $$\;J_{o}=qn\mu \frac{V}{d}$$

where q is the elementary charge, n is the thermally generated free carrier density, μ is the electron mobility, V is the applied voltage, and d is the thickness of the ZnO layer in the ZnO-nanofilm-based W/ZnO/ITO memory cell.

In the Child’s law conduction region, the current density $\;J_{c}$ can be expressed as

(4) $$\;J_{c}=\frac{9}{8}\varepsilon \mu \xi \frac{V^{2}\;}{d^{3}}$$

where ε is the dielectric constant of the ZnO layer, and ξ is the proportion of the free carrier density to the total carrier density. Obviously, the resistive switching behavior of the as-prepared W/ZnO/ITO memory cell switches from the HRS to LRS in the set process and then to HRS in the reset process, which might be consistent with the partial formation and rupture of conducting nanofilaments, respectively. Thus, the nonvolatile bipolar resistive switching behavior of the W/ZnO/ITO memory cell might be associated with the filamentary resistive switching mechanism.

The endurance performance of the as-prepared ZnO-nanofilm-based W/ZnO/ITO memory cell was recorded at 0.5 V for 100 successive cycles, as displayed in Figure 4c. It is clearly shown that both the LRS and HRS were highly stable and the resistance ratio between the HRS and the LRS exceeded 50, indicating the highly reproducible and reliable resistive switching capability of the ZnO-nanofilm-based W/ZnO/ITO memory cell. Figure 4d depicts the retention test of the ZnO-nanofilm-based W/ZnO/ITO memory cell. Obviously, the resistance ratio between the HRS and LRS can be stably maintained over 10^3^ s and without evident deterioration. For comparison, Table 1 tabulates the performance comparison of other ZnO-based memory devices [5,21,24,27,30,32,34,36,38,40,43,44,46,48,49,50,51,52,53,54,55]. The as-prepared ZnO-nanofilm-based W/ZnO/ITO memory cell in this work has a relatively higher resistance ratio of about two orders of magnitude and a facile preparation process, which demonstrates the promising potential of the as-prepared ZnO-nanofilm-based W/ZnO/ITO memory cell for applications in next-generation nonvolatile memory devices.

**Figure 5:**
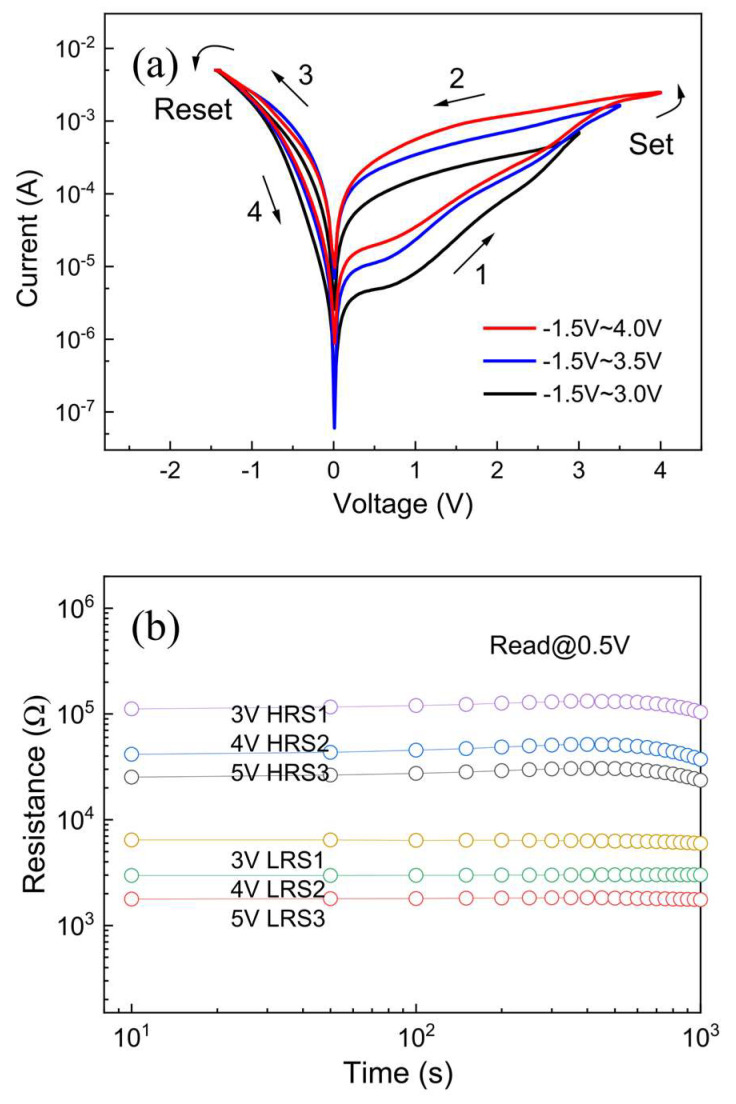
(**a**) The semi-logarithmic *I*-*V* curves and (**b**) retention capabilities of the W/ZnO/ITO memory cell under different set voltages.

To realize high-density nonvolatile memory applications, an effective strategy is to utilize the controllable multilevel resistive switching properties of the device itself. It was found that the multilevel resistive switching properties of the device can be obtained not only by regulating the reset voltages in the reset process, but also by adjusting the set voltages and compliance currents in the set process. Several previous reports have demonstrated that the multilevel resistive switching properties can be achieved by governing the compliance currents [3,7,25] and reset voltages [31,39] of the ZnO-based devices, but multilevel resistive switching properties were not observed by modulating the set voltages in the ZnO-nanofilm-based W/ZnO/ITO memory cell to date. Here, the multilevel resistive switching performances of the ZnO-nanofilm-based W/ZnO/ITO memory cell were achieved by modulating different set voltages while fixing the reset voltage at −1.5 V, as recorded in Figure 5a. It is appreciable that six distinguishable resistance states corresponding to three HRSs (HRS_1_, HRS_2_, and HRS_3_) and three LRSs (LRS_1_, LRS_2_, and LRS_3_) can be observed clearly, which are attributed to the partial formation of conductive nanofilament under different set voltages in the ZnO-nanofilm-based W/ZnO/ITO memory cell. The larger the set voltages applied to the ZnO-nanofilm-based W/ZnO/ITO memory cell, the more filaments were generated between the W top electrode and the bottom ITO electrode, resulting in the lower multilevel resistance states in both HRS and LRS. Figure 5b reveals the retention capabilities of the ZnO-nanofilm-based W/ZnO/ITO memory cell with multilevel resistance states, which demonstrates the stable and nonvolatile multilevel resistive switching properties. The resistance ratio between the HRS and the LRS can be adjusted from one to two orders of magnitude, maintained over 10^3^ s without evident deterioration, indicating the excellent potential for high-density nonvolatile memory applications.

**Figure 6:**
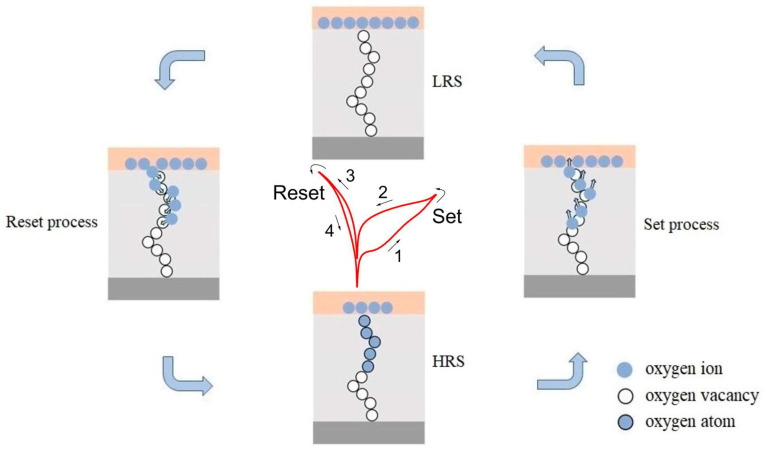
Schematic of the resistive switching mechanism of the W/ZnO/ITO memory cell.

As mentioned above, the nonvolatile resistive switching performance of the as-prepared ZnO-nanofilm-based W/ZnO/ITO memory cell might be assigned to the filamentary resistive switching mechanism modified by oxygen vacancies. During the set process shown in Figure 6, when the positive sweeping voltage is applied to the ZnO-nanofilm-based W/ZnO/ITO memory cell in the HRS, the oxygen ions drift upward and accumulate at the W top electrode, while the oxygen vacancies will migrate from the W top electrode to the bottom ITO electrode and develop into the metallic conductive nanofilaments across the wurtzite ZnO layer. Once the partial formation of conductive nanofilament occurs with the increasing applied voltage, the ZnO-nanofilm-based W/ZnO/ITO memory cell will transition from the HRS to LRS with the rise in current. Subsequently, the device will keep the LRS until a large enough negative sweeping voltage is applied, indicating the nonvolatile feature of the device. During the reset process, when the negative sweeping voltage is applied to the ZnO-nanofilm-based W/ZnO/ITO memory cell, the external electric field will drive the oxygen vacancies to move toward the W top electrode and recombine with oxygen ions at the W/ZnO interface, causing the partial rupture of the conductive nanofilaments. After that, the device recovers to the pristine HRS with the drop in current. Thus, the partial formation and rupture of conductive nanofilaments modified by oxygen vacancies are proposed to be responsible for the nonvolatile resistive switching behavior of the as-prepared ZnO-nanofilm-based W/ZnO/ITO memory cell.

Additionally, the multilevel resistive switching performances of the as-prepared ZnO-nanofilm-based W/ZnO/ITO memory cell were achieved by modulating different set voltages while fixing the reset voltage at −1.5 V. By applying higher set voltages, more oxygen vacancies will migrate toward the bottom ITO electrode and form more nanofilaments, which leads to the lower multilevel resistance states of the device in both HRS and LRS, suggesting the excellent potential for future high-density nonvolatile memory applications.

## 3. Experimental Details

All the used chemicals, including zinc acetate dihydrate (Zn(C_2_H_3_O_2_)·2H_2_O, 99%), isopropyl alcohol ((CH_3_)_2_CHOH, 99.7%), and ethanolamine (NH_2_(CH_2_) _2_ON, 99%) are of analytical grade and were used without further purification, and were purchased from the Sigma-Aldrich. The commercially available ITO (ITO, 7 Ω/square) substrates with a size of 1 cm × 2 cm were used for the epitaxial growth of the spin-coated ZnO nanofilms. The ITO substrates were cleaned ultrasonically for 30 min in a mixture composed of 30 mL of acetone, 30 mL of isopropanol, and 30 mL of deionized water, and then the cleaned ITO substrates were dried at room temperature.

The hexagonal phase ZnO nanofilms were directly prepared by a facile sol–gel spin coating method as follows. In brief, 0.5 mM of zinc acetate dihydrate was dissolved into a mixture containing 20 mL of isopropyl alcohol and 600 μL of ethanolamine. After constant stirring at 60 °C for 24 h, a yellow transparent mixture solution was generated, which was used as the ZnO precursor solution. The as-prepared ZnO precursor solution was spin coated onto the ITO substrates at 2000 rpm for 20 s. Then, the spin-coated nanofilms were dried at 60 °C for 5 min. After repeating the sol–gel spin coating process four times, the spin-coated ZnO precursor nanofilms were prepared. After that, the spin-coated ZnO precursor nanofilms were annealed in a muffle furnace at 500 °C for 10 min. Subsequently, the sol–gel spin coating and annealing processes were repeated again and the spin-coated ZnO nanofilms were obtained. The circular W top electrodes with a diameter of 5 μm in the W/ZnO/ITO memory cell were deposited on the as-obtained spin-coated ZnO nanofilms through the magnetron sputtering procedure.

The crystal phases and morphologies of the ZnO nanofilms were identified by using the X-ray diffraction (XRD, PANalytical PW3040/60, Cambridge, UK) with Cu Kα radiation (λ = 0.1541 nm) and Field-emission scanning electron microscopy (FESEM, FEI Nova NanoSEM 450, Lincoln, NU, USA), respectively. The chemical states of the ZnO nanofilms were confirmed using X-ray photoelectron spectroscopy (XPS, AXIS-ULTRA DLD-600W, Manchester, NH, USA) with monochromatic Al Kα radiation (hv = 1486.6 eV). The current–voltage (*I*-*V*) properties of the W/ZnO/ITO memory cell were tested using an Agilent B2901A semiconductor parameter analyzer. During the tests, the bias voltages of the ZnO-nanofilm-based W/ZnO/ITO memory cell were applied to the W top electrode with the bottom ITO electrode grounded, and a compliance current fixed at 5 mA was set to protect the device from irreversible breakdown.

## 4. Conclusions

In conclusion, hexagonal phase ZnO nanofilms with the growth axis perpendicular to the ITO substrate were synthesized using a facile sol–gel spin coating method. A hexagonal phase ZnO-nanofilm-based W/ZnO/ITO memory cell has been prepared for the first time. The as-prepared hexagonal phase ZnO-nanofilm-based W/ZnO/ITO memory cell possessed forming-free and tunable nonvolatile multilevel resistive switching behaviors with a relatively higher resistance ratio of about two orders of magnitude, which can be maintained over 10^3^ s and without obvious deterioration. The tunable nonvolatile multilevel resistive switching performances of the as-prepared W/ZnO/ITO memory cell were achieved by modulating the different set voltages and the resistance ratio between the HRS and the LRS which could be adjusted from one to two orders of magnitude. Furthermore, the carrier transport properties of the as-prepared ZnO-nanofilm-based W/ZnO/ITO memory cell were assigned to the Ohmic conduction mechanism in the low resistance state and the trap-controlled space-charge-limited current conduction mechanism in the high resistance state. In addition, the partial formation and rupture of conducting nanofilaments modified by the intrinsic oxygen vacancies in the as-prepared ZnO nanofilms were found to be responsible for the forming-free and tunable nonvolatile resistive switching behaviors of the as-prepared ZnO-nanofilm-based W/ZnO/ITO memory cell. This work suggests that the as-prepared W/ZnO/ITO memory cells may be a promising candidate for applications in future high-density nonvolatile memory devices.

## Figures and Tables

**Table 1:** Performance comparison of the ZnO-based memory devices.

Device Structure	V_set_/V_reset_ (V)	Preparation Process	R_HRS_/R_LRS_ Ratio	Retention	Reference
top-probe/α-Fe_2_O_3_/ZnO/bottom-probe	−0.55/−	Spin coating technique	~20	10^3^ s	[5]
Ag/ZnO/Pt	+1/−1	Magnetron sputtering	~10	10^3^ s	[21]
Ag/ZnO/Pt	~+2/~−0.5	Chemical vapor deposition	>50	10^3^ s	[24]
Pt/ZnO/Pt	+1.2/−1	Chemical vapor deposition	~7	10^4^ s	[27]
Cr/ZnO/Pt	~+0.5/~−0.5	Magnetron sputtering	~10	-	[30]
Pt/ZnO/Zn	−4/+5	Hydrothermal method	~10	10 s	[32]
Al/Si/Al_2_O_3_/(ZnO/Al_2_O_3_/Al)	+7/−7	Pulsed laser deposition	~10	10^3^ s	[34]
Pt/ZnO/TiN	~+1.25/~−1	Pulsed laser deposition	~2	-	[36]
Au/ZnO nanorods/AZO	−6/+7	Dip coating method	~10	-	[38]
Pt/ZnO/ITO	+1/−1	Cyclic voltammetry deposition	~50	3 × 10^2^ s	[40]
ITO/HfO_x_/ZnO/ITO	~−3/~+3	Magnetron sputtering	~10	10^4^ s	[43]
Cu/ZnO/ITO	+1/−1.7	Magnetron sputtering	~10	-	[44]
Ag/ZnO/Ag	~+1.6/~−2	Spin coating technique	<10	3.1 × 10^3^	[46]
Pt/ZnO NRL/ITO	+0.72/−0.59	Hydrothermal method	~10	10^3^ s	[48]
Ti/ZnO/Pt	~+2/~−1.5	Magnetron sputtering	~10	10^5^ s	[49]
Pt/ZnO nanowire/Pt	+0.5/−	Chemical vapor deposition	~1.5	0.9 × 10^2^ s	[50]
Ag/BaTiO_3_/γ-Fe_2_O_3_/ZnO/Ag	+3.1/−4.7	Co-precipitation method	~10	-	[51]
Pt/ZnO thin film/Pt	~−1.75/~+2	Magnetron sputtering	~10	10^3^ s	[52]
Au/ZnO/ITO	~+2.2/~−3.8	Magnetron sputtering	>10	-	[53]
Cr/ZnO/Pt–Fe_2_O_3_ NPs/ZnO/Cr	−7/+7	Dip coating method	~5	10^4^ s	[54]
Pt/ZnO_1−x_ NRs/ZnO TF/Pt	~+1.5/~−0.7	Chemical vapor deposition	40	10^4^ s	[55]
W/ZnO/ITO	+3/−1.5	Spin coating technique	50~10^2^	>10^3^ s	This work

## Data Availability

The data presented in this study are available on request from the corresponding author.

## Sample Availability

Samples are available from the author.
